# Ascariasis and hyperosmolar hyperglycemic state: a surprising ultrasound finding in the emergency department

**DOI:** 10.1186/s12245-017-0138-7

**Published:** 2017-03-21

**Authors:** Giles N. Cattermole, Jean-Paul Nzabandora

**Affiliations:** Emergency Department, Centre Hospitalier Universitaire de Kigali, University of Rwanda, Butare, Rwanda

**Keywords:** Ascariasis, Ultrasonography, Diabetes complications

## Abstract

**Background:**

We report the ultrasound finding of ascariasis in a patient with hyperosmolar hyperglycemic state (HHS). Although ascariasis is common in low-resource settings, there has been no previous report associating ascariasis with HHS.

**Case presentation:**

A 26-year-old Rwandan man was admitted to the emergency department in coma, with a glycemia of 600 mg/dl. He was resuscitated with fluids, intubated and ventilated, and treated with insulin and antibiotics. On day 3, an ascaris worm was passed via his nasogastric tube, and abdominal ultrasound revealed a heavy worm load. He was treated with albendazole and eventually made a full recovery.

**Conclusions:**

This is the first report of ascariasis as a potential cause of HHS, and we recommend that emergency practitioners consider early abdominal ultrasound in patients with hyperglycemic emergencies in areas with a high prevalence of ascariasis.

**Electronic supplementary material:**

The online version of this article (doi:10.1186/s12245-017-0138-7) contains supplementary material, which is available to authorized users.

## Background

*Ascaris lumbricoides* is an intestinal parasite common in many low-income countries, associated with poor sanitation [1]. Although over a billion people worldwide are affected, it is still considered a neglected tropical disease [2]. Adults with ascariasis are often asymptomatic, but massive worm loads can cause intestinal or biliary obstruction, especially in children. Complications of ascaris infestation also include pancreatitis, hepatic abscess, and encephalitis.

Rwanda has a high prevalence of ascariasis. The most recent nationwide study of over 8000 children found a prevalence of 38.6%. In some rural districts, this rises to over 50%. Despite treatment programs, transmission and re-infection continues throughout Rwanda [3].

We report the ultrasound findings of ascariasis in a patient with hyperosmolar hyperglycemic state (HHS). There has been no previous report associating ascariasis with HHS.

## Case presentation

A comatose 26-year-old Rwandan man was admitted to the emergency department (ED) at the University Teaching Hospital in Kigali, the capital city of Rwanda. He was hypoxic, tachypneic, and tachycardic, but normotensive, with a GCS of 6/15. Glycemia was 600 mg/dl (33.3 mmol/l). Initial treatment included fluid resuscitation, intubation, and ventilation. Temperature measurement and point-of-care blood gas analysis were not available in the department at that time. Urinary ketones were negative. A presumed diagnosis of HHS was made, and insulin infusion was started as well as broad-spectrum antibiotics. No beds were available in the intensive care unit, so he was managed for several days in the ED. There was no significant past medical or drug history.

Initial laboratory results included a mildly raised white cell count, with normal electrolytes. He was HIV-negative. There was no bacterial growth in blood cultures, CSF, or urine. Chest X-ray and CT brain with contrast were both normal.

**Fig. 1:**
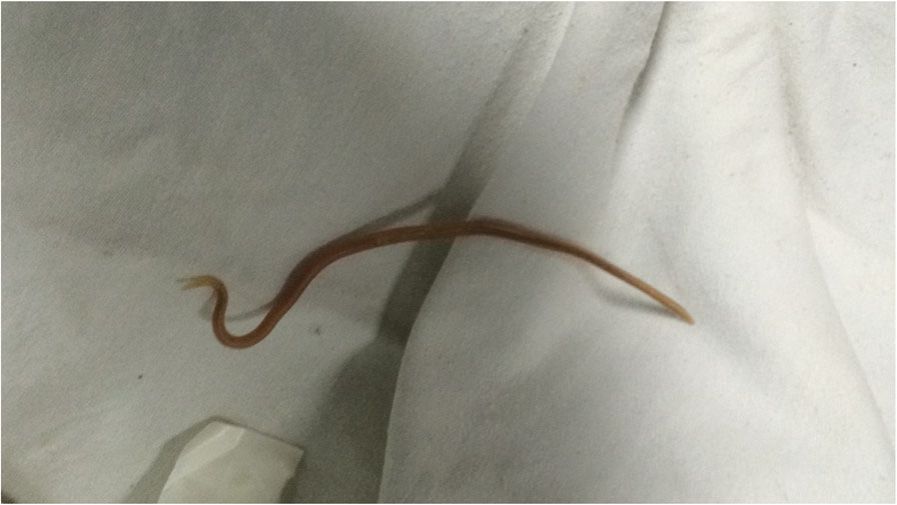
Ascaris worm

**Fig. 2:**
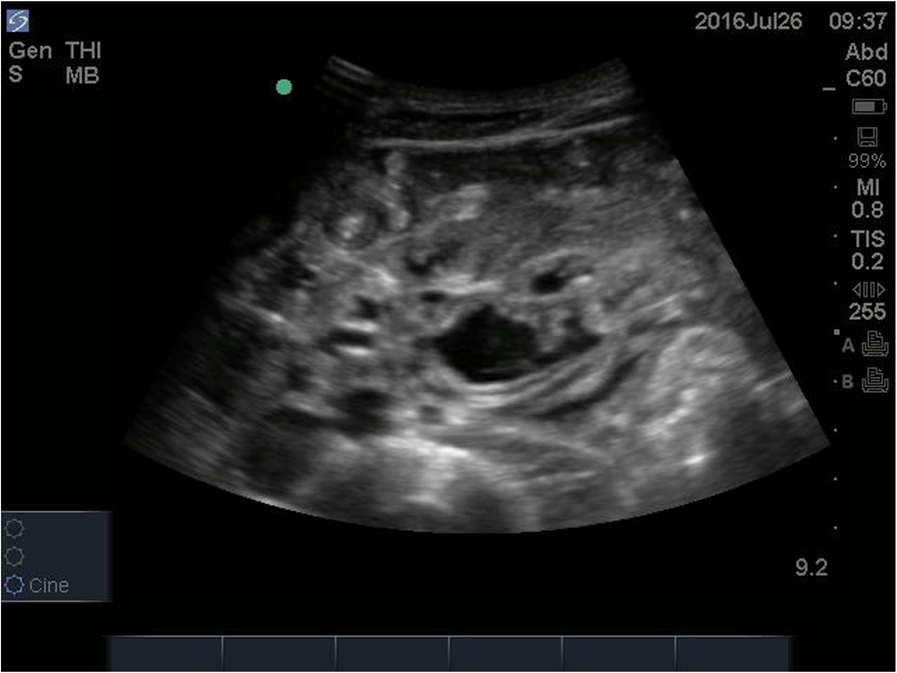
Abdominal ultrasound scan


On day 3, a 30-cm adult female ascaris worm was aspirated from his nasogastric tube (Fig. 1), and abdominal ultrasound revealed a heavy worm load (Fig. 2, Additional file 1: Video 1 and Additional file 2: Video 2): several intra-luminal echogenic tubular structures exhibiting slow curling movements were seen in the small bowel. He was treated with albendazole and made a full recovery. No other precipitant for his HHS was identified, and he was discharged with a new diagnosis of type 2 diabetes mellitus, on metformin and glibenclamide.

Additional file 1: Video S1. Abdominal ultrasound scan. (MP4 275 kb)



Additional file 2: Video S2. Abdominal ultrasound scan. (MP4 266 kb)


## Conclusions

HHS is most commonly precipitated by infection [4]. In this case, no infection was identified other than the heavy infestation with ascaris. We conclude therefore that this might have been the significant contributory factor in the patient’s development of HHS. It is however possible that the HHS was precipitated by another infection, which itself might have been secondary to ascariasis infestation. In our hospital, patients do not receive investigations until paid for in advance. Our patient had no access to money and depended on social services agreeing to fund each investigation. This meant that all our tests were significantly delayed, and it is possible that our failure to identify alternative infection was due to empiric treatment with broad-spectrum antibiotics. It is also possible that the HHS occurred de novo, independent of the ascariasis. However, whether ascariasis was the ultimate or penultimate cause of the HHS, or merely associated with it, it needed to be identified and treated.

Ultrasound has been recommended elsewhere as a helpful initial diagnostic tool to diagnose suspected ascariasis, especially where stool analysis for eggs is not available. Characteristic sonographic findings are described as a “winding highway” or “railway track” of parallel lines on longitudinal scans, or a “target” or “bull’s eye” appearance on transverse scans. Live worms show characteristic slow, pendular, non-directional movements [5], as we have demonstrated. In our case, we obtained clear images using the standard abdominal probe (3.5 MHz). However, better images might be obtained with a linear 5–10 MHz probe if the patient’s morphology permits [6]. Ultrasound can also demonstrate some of the complications of ascariasis, including free fluid from perforation, bowel obstruction, and hepatic abscess.

Because of the financial difficulties many patients have in paying for formal imaging, our department routinely uses bedside ultrasound for a wide spectrum of presentations. This case demonstrated its potential for identifying an important infection in a critically ill patient.

Our patient lived in Kigali, the capital of Rwanda, which has a lower prevalence of ascariasis (<20%), but his family home was in a high prevalence district (>50%) [3]. In settings like ours, emergency practitioners should have a high index of suspicion that the patient might be infected. Ascariasis infection should be treated even in isolation, but it is especially important to recognise and treat if it is a potential cause or an exacerbation of a life-threatening complication. We therefore advise that emergency practitioners consider early abdominal ultrasound in patients with hyperglycemic emergencies in areas with a high prevalence of ascariasis.

## Abbreviations

CSF: Cerebrospinal fluid
ED: Emergency department
GCS: Glasgow Coma Score
HHS: Hyperosmolar hyperglycemic state

## References

[1] Crompton DWT (2001). Ascaris and ascariasis. Adv Parasitol.

[2] Dold C, Holland CV (2011). Ascaris and ascariasis. Microbes Infect.

[3] Rujeni N, Morona D, Ruberanziza E, Mazigo HD (2017). Schistosomiasis and soil-transmitted helminthiasis in Rwanda: an update on their epidemiology and control. Infect Dis Poverty.

[4] Pasquel FJ, Umpierrez GE (2014). Hyperosmolar hyperglycemic state: a historic review of the clinical presentation, diagnosis, and treatment. Diabetes Care.

[5] Umetsu S, Sogo T, Iwasawa K, Kondo T, Tsunoda T, Oikawa-Kawamoto M, KomatsuH IA, Fujisawa T (2014). Intestinal ascariasis at pediatric emergency room in a developed country. World J Gastroenterol.

[6] Thapa NB. Ultrasound diagnosis of intestinal ascariasis. Glob J Med Res-D. 2014;14. http://medicalresearchjournal.org/index.php/GJMR/article/view/596/513.

